# A Case of Quinine Amblyopia1Read before the Surgical Section, Buffalo Academy of Medicine, October 4, 1898.

**Published:** 1898-11

**Authors:** J. C. Clemesha

**Affiliations:** Buffalo, N. Y., Assistant surgeon to the Buffalo Eye and Ear Infirmary


### A CASE OF QUININE AMBLYOPIA.[1]

By J. C. CLEMESHA, M. D., M. R. C. S., L. R. C. P., Buffalo, N. Y.,
Assistant surgeon to the Buffalo Eye and Ear Infirmary.

ON SEPTEMBER 15, 1898, I was asked by the Ladies' Committee at Tonawanda to examine the eyes of Private J. F. W., invalided from Camp Alger on account of an attack of typho-malaria fever. He gave me the following history :

About June 1, 1898, he left Camp Black for Camp Alger. During the first week in August, he was taken ill with fever, diagnosticated as typho-malarial, and was ill two weeks before being sent to the base hospital, where he remained three days. From there he was sent home.

While ill for the fourteen days at his company's quarters, he was given one capsule of quinine, containing 2½ grains, three times a day and his sight or hearing was not in any way affected thereby.

During the first thirty-six hours of his stay in the hospital, he was given two 5-grain capsules of quinine every two hours, thus making 180 grains absorbed into the system during the above period. The twenty-four hours following he was given one 5-grain capsule, morning, noon and night. Next day, on his way home, he endeavored to read a newspaper on the train, but found himself unable to read even the coarse print. His sight for distance was much deteriorated and he saw objects as through a mist, while surrounding lamp and gas flames he saw colored lights or a "halo." His eyes were somewhat inflamed and he suffered a good deal of pain, which seemed of a neuralgic character. His hearing had become defective and noises sounded in his ears like "the roar of a heavy sea." Besides these symptoms he complained much of dizziness, so that each time he stooped he felt as though he would fall upon his head. When seen by me, on September 15, 1898, his vision was :

$$\left.\begin{matrix} R \ \frac{20}{50} \ \text{Sn viii.} \\ L \ \frac{20}{70} \ \text{Sn viii.} \end{matrix}\right\} \bigcirc + \text{D. 1.00 sph. } \frac{20}{40} \ \text{Sn iv.}$$

There was a moderate injection of the conjunctiva, both palpebral and ocular. The pupils somewhat dilated, but reacted sluggishly to light and accommodation. Tension normal. The fields of vision were generally contracted, the left field slightly more than the right. On examination of the fundi, there was some pallor of the optic discs and the vessels seemed slightly smaller in diameter. There was no color blindness.

A pair of spectacles was ordered and five minims of liquor arsenicalis were ordered to be taken three times a day.

On September 22, 1898, his distant vision had improved a little, but not so for reading :

$$\left.\begin{matrix} R \ \frac{20}{50} \ \text{Sn viii.} \\ L \ \frac{20}{40} \ \text{Sn viii.} \end{matrix}\right\}$$

However the fields had much improved, being but a few degrees from what is considered as normal. His hearing is still deficient and the tinnitus has not decreased.

---

1. Read before the Surgical Section, Buffalo Academy of Medicine, October 4, 1898.

On October 12th his distant vision was normal:

R $\frac{20}{20}$ Sn ii. }
L $\frac{20}{10}$ Sn ii. }

Fields of vision normal and pupils reacted well to light and accommodation.

Knapp[1] has stated the following signs and symptoms of quinine amblyopia: Marked pallor, general weakness, twitching of the mouth and extremities. Total blindness and deafness, associated with loud tinnitus aurium. The pupils are widely dilated and do not react to light, but may to accommodation. The patient often loses consciousness to a greater or less extent and it may be that the blindness and deafness is not noticed for several days, because of the mental condition present. (It was so in this case, as his mind was supposed to be affected and the cerebral trouble). The ophthalmoscope shows an absolute anemia of the optic nerve and retina. The papilla is chalky white and no trace of a bloodvessel in that or the retina is seen.

The action of poisonous doses of quinine is undoubtedly due to a constriction of the peripheral vessels, producing an ischemia of the retinal and optic nerve vessels. If the blindness continues for a sufficient length of time, changes occur in the bloodvessels and an atrophy of the optic nerve and optic tract ensues.

Dr. Ward Holden, in a paper read before the American Ophthalmological Society, giving an account of his experimental researches on this subject, arrived at the following conclusions: In the first place, the effect of toxic doses of quinine caused spasm of the vessels and a lessened but not obliterated blood supply. As a result the inner layer of the ganglion cells and the nerve fibers broke down, but the cells of the inner nuclear layer were not visibly affected.

An ascending degeneration of the optic nerve fibers followed the retinal changes.

The prognosis is favorable as far as regards central vision. The damage to peripheral vision is likely to persist. When sight begins to return, most cases will be found to be color blind, completely or partially.

Rogers[2] thinks that incomplete ocular cinchonism is not rare and asserts that in an hour after twenty grains of quinine have been taken some accommodative paresis may be noticed in a goodly percentage of cases, and was also present in the above case.

---

1. Casey Wood, Annals of Ophthalmology, 1894
2. Ibid.

329 FRANKLIN STREET.

